# An Investigation of Virulence Genes of *Staphylococcus aureus* in Autologous Vaccines Against Sheep Mastitis

**DOI:** 10.3390/ani14223172

**Published:** 2024-11-06

**Authors:** Erminia Sezzi, Rita Fanelli, Deborah Gobbi, Paolo Scandurra, Valerio Mannucci, Isabella Usai, Giovanni Ragionieri, Ziad Mezher, Gianluca Fichi

**Affiliations:** 1 Istituto Zooprofilattico Sperimentale del Lazio e della Toscana M. Aleandri, UOT Lazio Nord, Strada Terme 4/a, 01100 Viterbo, Italy; erminia.sezzi@izslt.it (E.S.); rita.fanelli@izslt.it (R.F.); deborah.gobbi@izslt.it (D.G.); 2 Istituto Zooprofilattico Sperimentale del Lazio e della Toscana M. Aleandri, UOT Toscana Sud, Via Toselli 12, 53100 Siena, Italy; scandurrapaolo73@gmail.com (P.S.); valerio.mannucci@izslt.it (V.M.); isabella.usai@izslt.it (I.U.); giovanni.ragionieri@izslt.it (G.R.); ziad.mezher@izslt.it (Z.M.)

**Keywords:** *Staphylococcus aureus*, virulence genes, sheep mastitis

## Abstract

*Staphylococcus aureus* is well known to be the primary causal agent of clinical or subclinical mastitis in dairy sheep. The production of virulence factors allows *S. aureus* strains to cause mastitis. In the present study, 96 strains isolated from dairy sheep farms u*s*ed for the production of autologous vaccines were tested for enterotoxin and hemolysin genes by PCR. In addition, 14 strains isolated from half udders of ewes with subclinical mastitis belonging to a single farm were also tested for the same genes. The phylogenetic trees were constructed, and spatial analysis was performed. Overall, 20 gene patterns were identified, but 43.64% of the tested strains showed the same profile (*sec*+, *sel*+, *hla*+, *hld*+, *hlgAC*+). Considering only the enterotoxin genes, four profiles were identified while the evaluation of the hemolysin genes revealed the presence of 12 gene patterns. In the farm with subclinical mastitis, six gene profiles were found. Spatial analysis of the isolated strains and their virulence genes did not show a specific pattern. The present study highlights the importance of identifying and analyzing virulence genes of *S. aureus* strains involved in dairy sheep mastitis, and the presence of different strains in the same farm.

## Simple Summary

*Staphylococcus aureus* is a human and animal pathogen that can cause severe mastitis in sheep. The severity of this disease is influenced by several factors, among which the presence of virulence genes in the bacterium. In the present study, 110 strains, isolated from the milk of sheep with clinical mastitis south of Tuscany, Italy, and with subclinical mastitis in a farm in the same region, were investigated for enterotoxin and hemolysin genes’ presence through molecular analysis. The distribution of the profiles on this territory was analyzed. Twenty different profiles were found, but 43.64% of the tested strains showed the same profile. Considering the enterotoxin genes, four profiles were identified while using hemolysin genes’ presence, 12 genes patterns were found. Several different strains were detected in the farm with subclinical mastitis. Spatial analysis of the overall isolated strains did not show a specific distribution. However, the study highlights the importance of identifying and analyzing virulence genes of this bacterium involved in dairy sheep mastitis, and the presence of different strains in the same farm.

## 1. Introduction

Staphylococci are a major cause of clinical or subclinical mastitis in dairy sheep, and among them *Staphylococcus aureus* is well known to be the primary causal agent [1,2].

In small ruminants, especially sheep, *S. aureus* often causes severe mastitis, and it is the principal cause of gangrenous mastitis [3]. For this reason, small ruminants are often vaccinated against *S. aureus* with commercial vaccines, but in some countries, autologous vaccines are also used [4]. However, *S. aureus* can also cause subclinical mastitis in sheep, resulting in long-term infection that can evolve into chronic forms, with the consequent loss of milk production [5].

The severity of staphylococcal infections depends on the relationship between the host’s defense abilities and the strain’s virulence [6]. The production of virulence factors, such as toxins, antigens, and resistance proteins, allows *S. aureus* strains to cause mastitis [7].

Many virulence factors that allow the bacterium to adapt to the mammary gland environment [8] have been described in *S. aureus* (Table 1), and several of them have been identified in ruminant mastitis [8,9,10]. These virulence factors include more than 40 known exotoxins that can be classified into three groups based on their known functions: cytotoxins, superantigens, and cytotoxic enzymes [11]. After the adhesion phase, *S. aureus* produces hemolysins and exoenzyme exotoxins that allow the invasion and destruction of the mammary tissue, with the degradation of the epithelium of the cistern, duct, and alveoli [10].

Most of the staphylococcal enterotoxins (SEA, SEB, SECn, SED, SEE, and SEH) belong to staphylococcal pyrogenic exotoxin (PTSAgs) [9]. They have the ability to inhibit host immune responses to *S. aureus* [9], but SAgs were originally termed staphylococcal enterotoxins (SEs) for their ability to cause typical symptoms of *S. aureus* food poisoning, such as vomiting and diarrhea [15]. The α-, β-, γ-, and δ-hemolysins are identified as important virulence factors, produced respectively by *hla*, *hlb*, *hlg* and *hld* genes, which allow *S. aureus* to escape the host immune response and the invasion of the mammary tissue [28,29]. They create pores in host cell membranes or dissolve cell wall components, lysing erythrocytes [30]. Among them, α-hemolysin is considered one of the main pathogenicity factors of *S. aureus* with hemolytic, dermonecrotic, and neurotoxic effects [28]. All these genes can be used to characterize *S. aureus* strains. Numerous studies investigated virulence factors in bovine mastitis [31,32,33,34,35,36,37,38,39,40,41,42,43,44,45,46] and some of them also explored their geographical variability [8,47,48]. In contrast, the presence of *S. aureus* virulence genes has been less investigated in small ruminants [5,7,49,50,51,52,53], and the studies on their geographical distribution are rare [49].

From an epidemiological point of view, investigating the virulence factors’ profiles of *S. aureus* isolates involved in the etiology of mastitis is highly relevant, provided that the distribution of the bacterial strains in a given territory is constantly monitored [48]. In parallel, conducting such investigations on isolated *S. aureus* strains from sheep herds with mastitis could provide relevant information for the development of vaccines [10].

The principal aim of the present study was to characterize *S. aureus* strains, collected during sheep mastitis episodes in a defined territory and used to produce autologous vaccines, by investigating the toxins’ patterns and their geographical distribution to deepen the knowledge on the circulating genetic lineages among the sheep population with mastitis. A second aim was the characterization of strains isolated from a sheep herd with confirmed subclinical mastitis due to *S. aureus* to investigate the toxins’ patterns of this bacterium.

## 2. Materials and Methods

### 2.1. S. aureus Strains

This study was carried out on 96 strains of *S. aureus* conserved at −80 °C in the microbank of the Laboratory for vaccine production of Istituto Zooprofilattico Sperimentale del Lazio e della Toscana M. Aleandri, Siena, Tuscany, Italy. The strains were isolated in sheep mastitis outbreaks of herds in Grosseto and Siena provinces, Tuscany, Italy and used to produce autologous vaccines. In addition to the 96 strains, 14 strains of *S. aureus*, isolated in 2022 from half udders of ewes with subclinical mastitis in a single farm in Grosseto province, during a *S. aureus* eradication program, were conserved under the same condition and included in the study. The strains were revitalized in brain heart infusion broth (BHI) overnight at 37 °C and tested for vitality and pureness on sheep blood agar after incubation at 37 °C for 24 h. Three ATCC strains of *S. aureus*, 23235, 19095, 70699, were used as positive control, while the ATCC 12228 of *S. epidermidis* was used as negative control.

### 2.2. Strains Isolation and Identification

The *S. aureus* strains were isolated between 2019 and 2023 from milk samples of sheep with clinical mastitis (Table S1), namely udder or milk alterations. Isolation was conducted by of the Veterinary Diagnostic laboratories located in Grosseto and Siena of the Istituto Zooprofilattico Sperimentale del Lazio e della Toscana M. Aleandri, following standard procedures [54]. Briefly, ten microliters of milk sample were plated onto blood agar medium through a sterilized loop and incubated at 37 °C under aerobic conditions. After 24 h, developed bacterial colonies were examined for taxonomical analyses. For this purpose, bacterial colonies morphologically characteristic for *S. aureus* on blood agar plates were plated on Baird Parker Agar Base + RPF (BP+RPF) and tested for catalase. Catalase-positive colonies with typical opacity halo on BP+RPF medium were biochemically identified at species level as *S. aureus* using API 20 STAPH micro-method galleries (BioMereux, Craponne, France).

### 2.3. DNA Extraction and Genes Amplification

To confirm the *S. aureus* identification, all isolates were screened by polymerase chain reaction (PCR) for the 23S, coagulase gene (*coa*), and *nuc* genes. DNA was extracted using the QIAamp DNA Mini kit following the instruction provided by the manufacturer. Briefly, a colony picked up from culture plate with an inoculation loop was suspended in 180 µL of buffer ATL (supplied in the kit). The extraction continued with the protocol for isolation of genomic DNA from Gram-positive bacteria, which involves three incubations at 37 °C (30 min), 56 °C (30 min), and 95 °C (15 min). Purification and elution followed the protocol for DNA purification from tissue. DNA of the reference samples was isolated from reference bacterial strains (ATCC 19095, ATCC 23235, ATCC 700699—*Staphylococcus aureus*, ATCC 12228 *Staphylococcus epidermidis*) following the same instruction. The genes investigated were the staphylococcal enterotoxin producing genes (*sea*, *sec*, *sed*, *seg*, *seh*, *sei,* and *sej*) and hemolysin genes (*hla*, *hlb*, *hld,* and *hlgAC*).

Extracted DNA was amplified using one multiplex PCR end point; each reaction contained 25 μL of Qiagen Multiplex Master Mix (Qiagen, Hilden, Germany), 1 μL of each primer (except the pair *Sei* where 1.5 μL) at 20 pmol/μL concentration and 1 μL of DNA template in 50 μL of total volume (Table 2). Thermal cycling was performed in a GeneAmp PCR System 9700 (Applied Biosystems, Foster City, CA, USA). For 23S, CoA, nuc and enterotoxin genes, thermal cycling consisted of an initial 15 min denaturation step at 95 °C, 30 cycles of 30 s at 94 °C, 1:30 min at the annealing temperature 57 °C, and 1:30 min extension step at 72 °C followed by 10:00 min at 72 °C [55]. For *hla* and *hld* genes, there was an initial denaturation step at 94 °C for 5 min, 35 cycles of 94 °C for 30 s, 59°C for 60 s, and 72 °C for 1 min, and a final extension step at 72 °C for 10 min, and for *hlb* an initial same step of denaturation followed by 45 cycles of 94 °C for 30 s, 65 °C for 30 s, and 72 °C for 30 s and same step of extension [56]. The *hlgAC* gene was amplified with a thermal cycling consisting of an initial 5 min denaturation step at 94 °C, 35 cycles of 30 s at 94.8 °C, 30 s at the annealing temperature 49.7 °C, a 1:30 min extension step at 72 °C followed by 7 min at 72 °C [42]. The three ATCC strains of *S. aureus* were used as positive control while the ATCC strain of S. epidermidis was used as negative control during PCR amplification of the investigated virulence factors.

The amplified PCR products were visualized by standard gel electrophoresis in a 4% agarose (Nippon Genetics, Düren, Germany) gel stained with nucleic acid gel stain (Midori Green Advance DNA stain) and molecular size marker (50 bp, Nippon Genetics).

### 2.4. Phylogenetic Analysis

Virulence gene profiles were analyzed as binary data [46] using the maximum likelihood method [58] by the Kimura 2-parameter model with 1000 bootstrap replicates by MEGA software (Version 11).

### 2.5. Spatial and Statistical Analysis

Excel spreadsheets (Version 2409 Excel^®^ Microsoft^®^, 2016) were used to create a dataset, which included all the relevant information about the investigated strains. Simple descriptive statistical analysis was performed by calculating the frequencies and confidence intervals of the strains’ profiles and relative virulence genes. The geographic information system software QGIS (Version 3.24.1) was used to create the distribution maps.

## 3. Results

All the analyzed strains tested positive for the 23S gene, and *coa* and *nuc* genes, confirming the *S. aureus* identification. Regarding the enterotoxin genes, the presence of *sea*, *sed,* and *sej* genes was not detected in any strain (Table 3). The *sec* and *sel* genes were detected in 74.55% of analyzed strains (82 strains), while *seg*, *seh*, and *sei* genes were observed in only one strain (0.91% of prevalence). The *hld* hemolysin gene was observed in 89.09% of the strains (98 strains), followed by *hlgAC* with a prevalence of 87.27% (96 strains), and *hla* 81.82% (90 strains). Only the 15.45% of the strains tested positive for the *hlb* gene. 

Considering all the 110 strains analyzed, 20 virulent gene profiles were found (SA1-SA20) (Table 4). The most common profiles were SA16 (*sec*+, *sel*+, *hla*+, *hld*+, *hlgAC*+) with a prevalence of 43.64 % and SA4 (*hla*+, *hld*+, *hlgAC*+) with a prevalence of 17.27%. 

**Figure 1:**
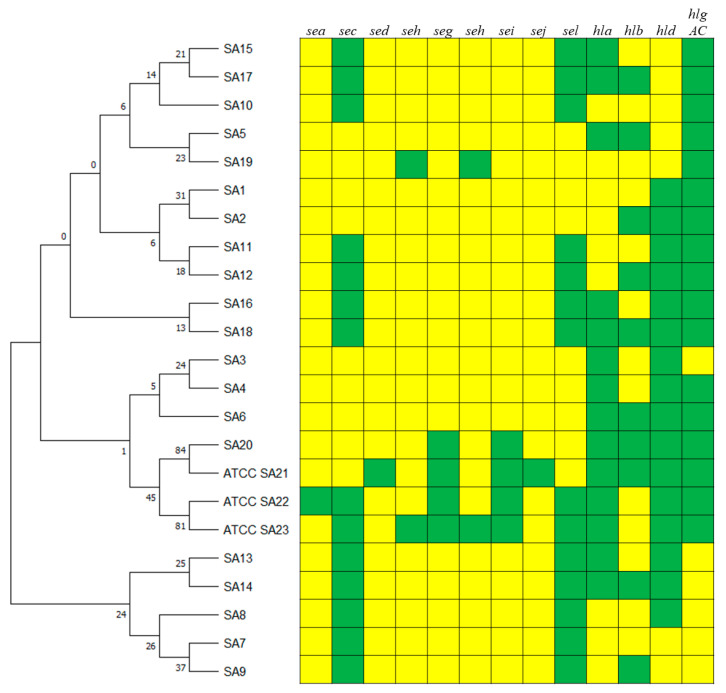
Analysis of 20 virulence gene profiles (SA1–SA20) of 110 *Staphylococcus aureus* isolates from sheep with clinical and subclinical mastitis and ATCC strain profiles. The dendrogram was generated based on the presence/absence of virulence genes using the maximum likelihood method by the Kimura 2-parameter model with 1000 bootstrap replicates. Branches corresponding to partitions reproduced in less than 50% bootstrap replicates are collapsed. The percentage of replicate trees in which the associated taxa clustered together in the bootstrap test are shown next to the branches. Green boxes indicate the presence and yellow boxes the absence of the corresponding virulence genes.

The phylogenetic analysis evidenced that SA1, SA2, SA5, SA10, SA11, SA12, SA15, SA16, SA17, SA18, and SA19 clustered together (Figure 1). The ATCC strains (ATCC 23235, 700699, 19095 called respectively SA ATCC21, SA ATCC22, SA ATCC23) clustered with SA3, SA4, SA6, and SA20, while the last strains SA7, SA8, SA9, SA13 and SA14 clustered together.

When only the enterotoxin genes were considered, four profiles were observed (SAET1-SAET4) (Table 5), but SAET 2 (*sec*+, *sej*+) and SAET1 (no virulence genes) were the most frequent profiles with a prevalence of 74.55% and 23.64%, respectively, while just one sample resulted for the profile SAET3 (*seh*+) and SAET4 (*seg*+, *sei*+).

**Figure 2:**
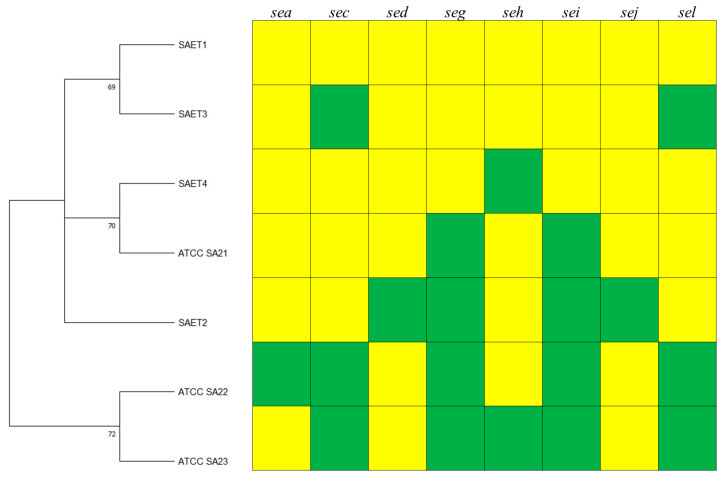
Analysis of four enterotoxin gene profiles (SA1–SA20) of 110 *Staphylococcus aureus* isolates from sheep with clinical and subclinical mastitis and ATCC strain profiles. The dendrogram was generated based on the presence/absence of virulence genes using the maximum likelihood method by the Kimura 2-parameter model with 1000 bootstrap replicates. Branches corresponding to partitions reproduced in less than 50% bootstrap replicates are collapsed. The percentages of replicate trees in which the associated taxa clustered together in the bootstrap test are shown next to the branches. Green boxes indicate the presence and yellow boxes the absence of the corresponding virulence genes.

The four enterotoxin gene profiles clustered with SA ATCC21, while the SA ATCC22, SA ATCC23 clustered together (Figure 2).

**Figure 3:**
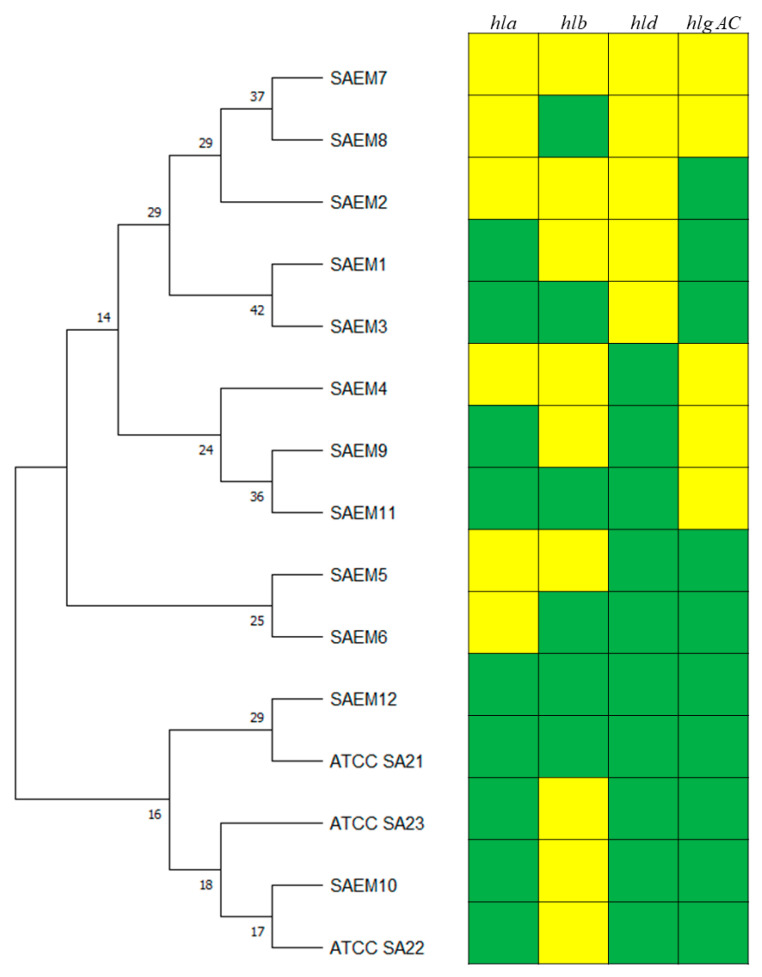
Analysis of 12 hemolysin gene profiles (SA1–SA20) of 110 *Staphylococcus aureus* isolates from sheep with clinical and subclinical mastitis and ATCC strain profiles. The dendrogram was generated based on the presence/absence of virulence genes using the maximum likelihood method by the Kimura 2-parameter model with 1000 bootstrap replicates. Branches corresponding to partitions reproduced in less than 50% bootstrap replicates are collapsed. The percentages of replicate trees in which the associated taxa clustered together in the bootstrap test are shown next to the branches. Green boxes indicate the presence and yellow boxes the absence of the corresponding virulence genes.

The analysis of the hemolysin genes revealed 12 profiles (Table 6). Most of the profiles clustered in the same group while SAEM10 clustered with ATCC SA22 and ATCC SA23 and SAEM12 clustered with ATCC SA21 (Figure 3). The most representative profile (60.91% of the strains) was SAEM10 (*hla*+, *hld*+, *hlgAC*+).

The 14 strains of *S. aureus*, isolated from half udders of ewes with subclinical mastitis at the same farm during a *S. aureus* eradication program, corresponded to six different virulence gene profiles (SA 7, SA9, SA 10, SA11, SA16, SA17). In this case, the most identified profile was SA16 (46.82%), followed by SA10 (21.43%) and SA11 (14.29%) (Table 6). The enterotoxin gene profiles were the same for all isolated strains (SAET 2: *sec*+, *sel*+), but not for the hemolysin genes that showed six profiles: SAEM 1 (no hemolysin genes), SAEM3 (*hlb*+), SAEM4 (*hlgAC*+), SAEM5 (*hlgAC*+, *hld*+), SAEM 10 (*hla*+, *hlgAC*+, *hld*+), and SAEM 11 (*hla*+, *hlb*+, *hlgAC*+). The frequency of the profiles was the same as the corresponding profiles when all genes were considered (Table 7). Not all profiles clustered together (Figure 1 and Figure 3).

**Figure 4:**
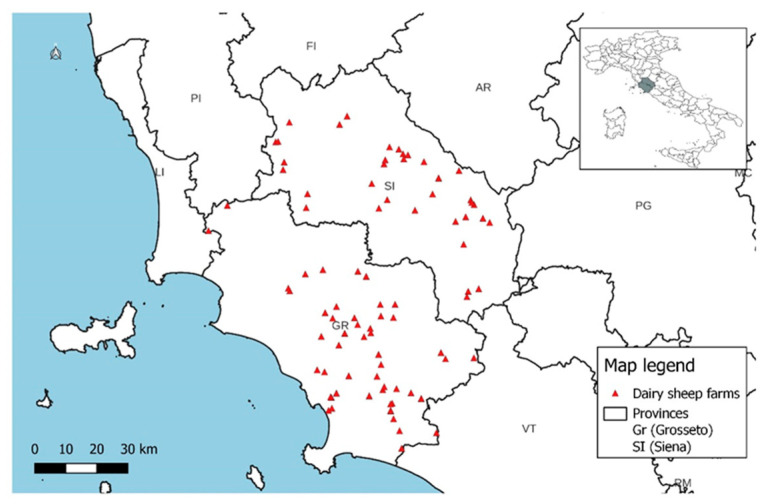
Position of investigated dairy sheep farms.

The isolated strains were collected from ewes reared in 86 dairy sheep farms located in the provinces of Siena (36/86; 41.86%) and Grosseto (50/86; 58.14%) in central Italy (Figure 4). These provinces are located in the southern part of the region of Tuscany, which is considered the fourth Italian region in terms of dairy sheep herds (219.424; 6.95%) after Sardinia, Sicily, and Lazio (data retrieved from the national farms’ registry). In Tuscany, dairy sheep farming is mainly concentrated in these two provinces (171.105; 77.97%) because of the land’s suitability for extensive sheep farming.

**Figure 5:**
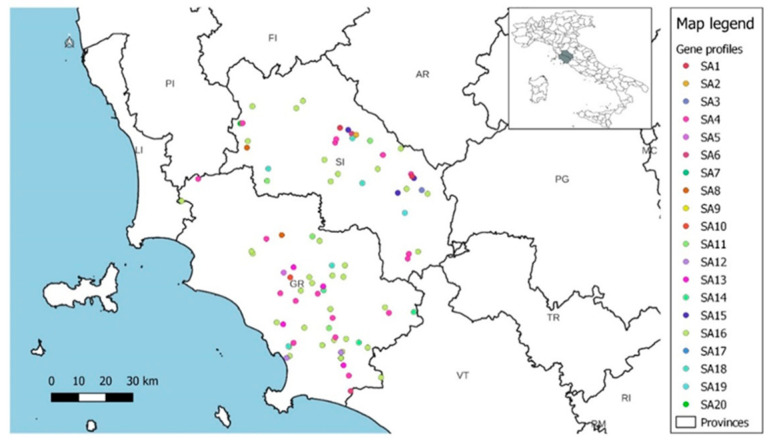
Spatial distribution of gene profiles in dairy sheep farms.

**Figure 6:**
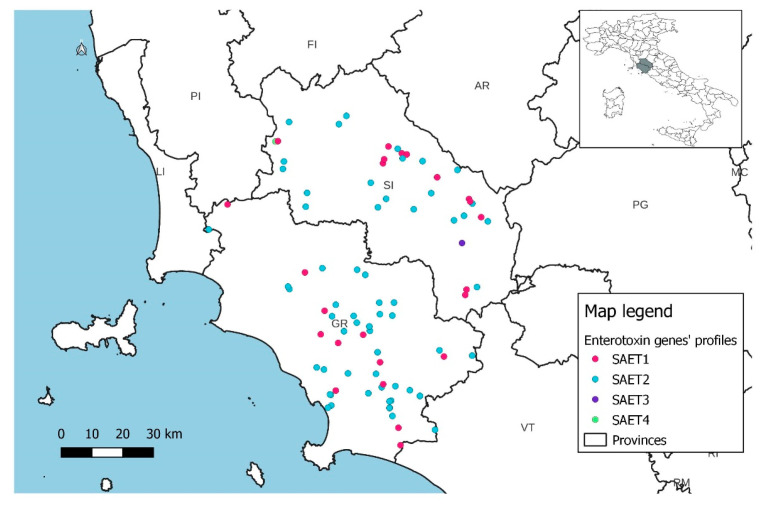
Spatial distribution of enterotoxin gene profiles in dairy sheep farms.

**Figure 7:**
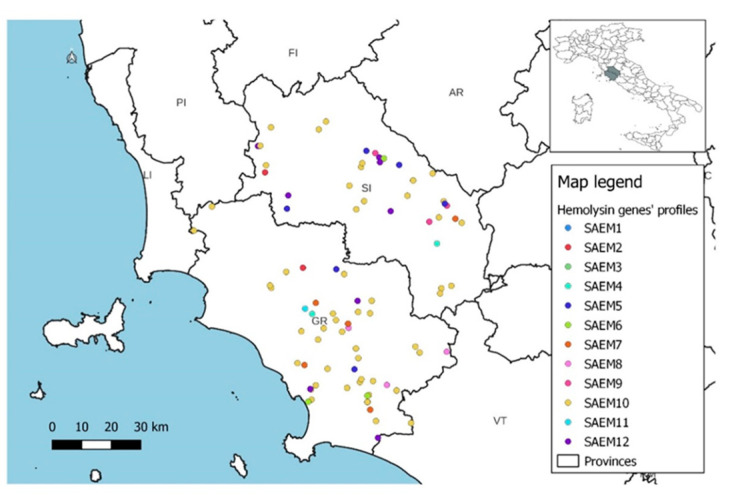
Spatial distribution of hemolysin gene profiles in dairy sheep farms.

Spatial distribution of the gene profiles and hemolysin gene profiles did not show the presence of specific patterns but only reflected the frequency of the various profiles within each province (Figure 5, Figure 6 and Figure 7).

## 4. Discussion

Very few studies have investigated the virulence gene profiles of *S. aureus* in sheep mastitis and its distribution in a geographic area. Considering the possible severity of this udder infection in sheep and the use of commercial and autologous vaccines, more study will be recommended. In the present study, enterotoxin and hemolysin genes were investigated in 96 strains, isolated in clinical mastitis, and distributed in a specific area, and 14 strains were isolated during an eradication program in sheep subclinical mastitis.

There is no consensus on analysis of virulence genes of *S. aureus* to investigate the relationship with clinical mastitis in cattle. Several studies suggested that enterotoxin genes act as virulence genes and they were used to classify some strains isolated in bovine subclinical mastitis and to correlate them with clinical observation [32,35,36]. The presence of *S. aureus,* which produces these toxins in the milk, can also have severe consequences for human health because these toxins remain stable in milk, causing food poisoning in humans [5].

Using RS–PCR genotyping of the 16S–23S rRNA intergenic spacer region, Fournier and collaborators associated some genotypes with the presence of some enterotoxin genes [35]. Particularly, genotype B (GTB) was characterized by the presence of the *sea*, *sed*, and *sej* genes, and genotype C (GTC) by *sec* and *seg*. The remainder were grouped as other genotypes (GTOG) because they were rarely found [35]. In our study, no profiles corresponded to GTB or GTC. The 74.55% of our strains had *sec* and *sel* gene profiles, but no *sed* gene was detected, and one strain showed the *seg* gene but in association with *sei*. However, in the literature, some studies on bovine mastitis found *seg* and *sej* genes as the most frequent genes in *S. aureus* [59] while others found *sed*, *seg,* and *sei* [60], or *sea,* as in a study on 238 *S. aureus* isolates from cow milk in two China regions [59]. The comparison with enterotoxin gen’ profiles in sheep is very difficult because the same genes were not amplified in the few studies published in literature. In a study on *S. aureus* isolated from bulk-tank milk samples of goat and sheep, the only gene in the strains isolated in 30 ewes’ milk was *sec* [53]. Strains positive for *seg* and *sei* genes’ presence were found in 18 goat milk samples, while no strain positive for *sec* and *sej* was found [53]. In another study on 20 *S. aureus* isolated from sheep milk samples with subclinical mastitis, *sea* and *seb* were observed in combination in three strains, *sea* and *sec* in one strain, and *sec* alone in another strain, but only *sea*, *seb*, *sec,* and *sed* enterotoxin genes were amplified [5]. A study conducted in Italy evidenced the combination of *seb* and *sec* as the most isolated genetic profile in *S. aureus* from sheep milk in Italy, but *sec* was the most frequent gene [61], as it was in a study of sheep milk in Algeria [62]. Another study conducted in Greece confirmed the *sec* profile as the most frequent in *S. aureus* strains from sheep milk, and a *seg-* and *sej-* positive profile was found in two strains [7]. In a study in Turkey, *sel*l had the highest frequency in sheep milk *S. aureus* strains, followed by *sec*, and the *sec* and *sel*l profile was the unique combination found [50]; additionally, *sec* and *sel* were the most detected genes in Switzerland [63].

Few studies investigated hemolysin genes in *S. aureus* strains isolated from mastitis milk of dairy species and there is no consensus on the genes found in the strains, as for enterotoxin genes. Aslantaş et al. (2022) [50] observed a frequency of *hla*, *hlb*, *hld,* and *hlg2* of 100, 95.2, 98.4, and 61.3%, respectively, in strains obtained from mastitic sheep milk. In another study on *S. aureus* strains from bovine and goat milk, the frequencies of *hla* and *hlb* were lower in goat strains (*hla* 51.85%, *hlb* 48.15%) respect to bovine strains (*hla* 95.93%, *hlb* 93.50%) [49]. In contrast, Moraveij et al. (2014) reported the presence of *hlb* only in one of 20 isolates of *S. aureus* from bovine mastitis milk, while the majority of the strains showed *hla* and *hld*, and no strains had the *hlg* gene [64]. In a study on 229 bovine *S. aureus* isolates in Belgium *hla*, *hlb*, *hld*, and *hlg* showed respectively a frequency of 98.7, 99.1, 100.0, and 78.6% [42]. In our study, the frequency of *hla*, *hld,* and *hlgAC* was 81.82, 89.09, and 87.27%, respectively, while the *hlb* gene was found with a lower frequency (15.45%). In contrast, a study conducted in Italy on strains from mastitis sheep milk found a higher frequency of *hlb*-positive strains (34.9%) and a lower frequency of *hlg* (2.7%) [61]. However, we observed a greater variability of the frequency of hemolysin genes in *S. aureus* strains than enterotoxin genes, with 12 and 4 different profiles, respectively. Particularly, in the farm with subclinical mastitis, all the strains showed the same profiles for enterotoxin genes, while six hemolysin gene profiles were observed. The diversity of toxin profiles of strains that cause mastitis is the major obstacle in the development of an effective vaccine [10], and the presence of several strains in the same farm could be a further obstacle.

In human isolates, it has been observed that the prevalence of hemolysin genes in multiple antibiotic-resistant strains was higher than in susceptible strains [65]. For this reason, the identification of isolates with hemolytic genes in mastitis milk samples could have a great importance [65]. However, the level of expression of genes during *S. aureus* infection is reported to be profoundly different from that of in vitro study [10], and the presence of virulence genes does not always correspond with the toxins’ expression [29]. We found high prevalence of *hla*, *hld*, and *hlg*AC, but further studies should be conducted to investigate the expression of these hemolysin genes during sheep *S. aureus* mastitis to understand their role in this disease.

It is also important to implement the spatial analysis of gene profiles to identify specific trends and geographical patterns over time. In this study, no specific trend or pattern was detected but profiles were apparently randomly distributed. This might be due to the small number of the analyzed strains in contrast with the prevalence of staphylococcal sheep mastitis or to the considered genes in the present study. However, in a previous study on spatial distribution of the virulence profiles of *S. aureus* strains isolated from cow and goat milk samples in three different geographical regions of the state Pernambuco, Brazil, no pattern in the frequency was observed by geographical area [49].

Therefore, the collection and analysis of further strains is encouraged to reach an adequate level of representativeness of the dairy sheep population in Tuscany, while the increase of the number of amplified genes could identify different profiles with a spatial pattern.

The antibiotic treatment of small ruminant mastitis is widely spread [66]. This has led to the development of bacterial resistance, and the presence of methicillin-resistant *S. aureus* (MRSA) strains in animals with the possibility of transferring resistance genes to human strains is an emerging problem [10]. In addition, the use of antibiotics for treating mastitis can lead to residues in milk with risks for human health [66]. The prevention of *S. aureus* mastitis through vaccination could be a valid alternative to antibiotic treatment [10]. However, the development of a vaccine against *S. aureus* is a challenge because of its complex pathogenesis, which involves numerous virulence factors [67]. The best vaccine should have an array of virulence factors [68]. The commercially available vaccines against *S. aureus* mastitis are unable to provide a complete protection [66], for this reason, autologous vaccines, which contain whole bacterial cells, are commonly used in dairy ruminant species [69], and some studies showed the benefit of the use of these vaccines in cow herds [69]. The autologous vaccine is often used in combination with commercial vaccines, especially in those cases where the latter did not provide sufficient protection against *S. aureus* and a consequent outbreak in the herd has occurred. The study of virulence factors of the strains used to produce these autologous vaccines, as in our study, could help to identify the virulence factors involved in this economically important disease and to develop an effective vaccine for this species. In the present study, the presence of staphylococcal enterotoxins and hemolysins was investigated, but the number of possible virulence factors involved in mastitis is higher and further study should be conducted to extend the number of investigated genes in the studied strains. However, most of the autologous vaccines are produced using a single clonal type of bacteria isolated from farms [70]. In contrast, we evidenced that more than one strain of *S. aureus* could be present on the same farm. The investigation of this aspect could help to make autologous vaccines more effective.

## 5. Conclusions

Considering the production of autologous vaccines from strains isolated by small ruminant mastitis in several countries, the study of virulence genes in these strains could help to develop a more efficient vaccine against *S. aureus* mastitis. In addition, six different hemolysin gene profiles were observed on the same farm with subclinical mastitis. This highlights the importance of testing the entire herd in cases of mastitis due to *S. aureus* because of the potential concomitant presence of several strains with different virulence characteristics and antibiotic susceptibilities.

## Figures and Tables

**Table 1:** Virulence factors of *Staphylococcus aureus* reported in literature.

Virulence Factors	Reference
Collagen-binding protein (Cna)	[12]
Fibronectin-binding proteins (FnBPA, FnBPB)	[12]
Clumping factors (ClfA, ClfB)	[12]
Serine–aspartate repeat proteins (Sdr A, Sdr B, Sdr C)	[12]
Iron-regulated surface determinant proteins (IsdA, IsdB, IsdH)	[12]
*S. aureus* surface protein (G SASG)	[12]
*S. aureus* capsular polysaccharides (13 serotypes)	[12]
Staphylokinase (SAK)	[12]
Extracellular fibrinogen binding protein (Efb)	[12]
Chemotaxis inhibitory protein of *S. aureus* (CHIPS)	[12]
Staphylococcal complement inhibitor (SCIN)	[12]
Formyl peptide receptor-like-1 inhibitory protein (FPRL1)	[12]
Extracellular adherence protein (Eap)	[12]
Hemolysins (αHL, βHL, δHL, γHL: HlgAB and HlgCB)	[13,14,15]
Leukocidins (LukAB, LukED, LukMFʹ, LukPQ)	[13,14,16]
Panton–Valentine leukocidins (LukSF–PV)	[17,18]
Hyaluronidase (hysA)	[13]
Staphylokinase (Sak)	[13]
Lipase (SAL1, SAL2)	[19]
Nuclease (Nuc)	[13]
Staphyloxanthin (STX)	[13]
Clumping factors (ClfA, ClfB)	[13]
Extracellular matrix protein (Emp)	[20]
Fibronectin binding proteins (FnBPA, FnBPB)	[21,22]
Fibrinogen binding protein (Efb)	[14,22,23]
Fibronectin-binding protein (FbpA)	[14]
Serine-aspartate repeat proteins (SdrC, SdrD, SdrE)	[22,24]
Anchored collagen adhesin (Cna)	[22]
Staphylococcal enterotoxins (SEA, SEB, SEC1, SEC2, SEC3, SED, SEE, SEG, SEH, SEI, SElJ, SEK, SEL, SEM, SEN, SEP, SEQ, SER, SES, SET, SEU, SElW, SEV, SElX, SelY)	[25]
Toxic shock syndrome toxin (TSST-1)	[15]
Exfoliative toxins (ETA, ETB, ETC, ETD)	[15,26]
Epidermal cell differentiation inhibitor exotoxins (EDIN-A, EDIN-B, EDIN-C)	[27]
Phenol-soluble modulins (PSMα, PSMβ, PSMγ)	[15]

**Table 2:** Primer and amplicon sizes of the target genes of *S. aureus*.

Target Gene	Oligonucleotide Sequence (5′-3′)	Amplicon Size (bp)	Reference
*23S rRna*	F: AGC TGT GGA TTG TCC TTT GG	499	[55]
	R: TCG CTC GCTBCAC CTT AGA AT		
*coa*	F: CCG CTT CAA CTT CAG CCT AC	204	[55]
	R: TTA GGT GCT ACA GGG GCA AT		
*nuc*	F: AGT TCA GCA AAT GCA TCA CA	400	[55]
	R: TAG CCA AGC CTT GAC GAA CT		
*sea*	F: TAA GGA GGT GGT GCC TAT GG	180	[55]
	R: CAT CGA AAC CAG CCA AAG TT		
*sec*	F: ACC AGA CCC TAT GCC AGA TG	371	[55]
	R: TCC CAT TAT CAA AGT GGT TTC C		
*sed*	F: TCA ATT CAA AAG AAA TGG CTC A	339	[55]
	R: TTT TTC CGC GCT GTA TTT TT		
*sej*	F: GGT TTT CAA TGT TCT GGT GGT	306	[55]
	R: AAC CAA CGG TTC TTT TGA GG		
*sel*	F: CAC CAG AAT CAC ACC GCT TA	240	[55]
	R: CTG TTT GAT GCT TGC CAT TG		
*seg*	F: CCA CCT GTT GAA GGA AGA GG	432	[55]
	R: TGC AGA ACC ATC AAA CTC GT		
*seh*	F: TCA CAT CAT ATG CGA AAG CAG	463	[55]
	R: TCG GAC AAT ATT TTT CTG ATC TTT		
*sei*	F: CTC AAG GTG ATA TTG GTG TAG G	529	[57]
	R: CAG GCA GTC CAT CTC CTG TA		[55]
*hla*	F: CTGATTACTATCCAAGAAATTCGATTG	209	[56]
	R: CTTTCCAGCCTACTTTTTTATCAGT		
*hlb*	F: GTGCACTTACTGACAATAGTGC	309	[56]
	R: GTTGATGAGTAGCTACCTTCAGT		
*hld*	F: AAGAATTTTTATCTTAATTAAGGAAGGAGTG	111	[56]
	R: TTAGTGAATTTGTTCACTGTGTCGA		
*hlgAC*	F: AATTCATTTGTTACACCGAATG	1245	[42]
	R: GCCATCGCATAGCTTTAACA		

**Table 3:** Prevalence and 95% confidence interval (95% C.I.) of tested genes in 110 strains of *S. aureus* isolated from sheep with mastitis.

Genes	Number	Frequence (%)	95% C.I. (%)
*sea*	0	0	0.00–0.34
*sec*	82	74.55	65.35–82.37
*sed*	0	0	0.00–0.34
*seg*	1	0.91	0.00–4.96
*seh*	1	0.91	0.00–4.96
*sei*	1	0.91	0.00–4.96
*sel*	82	74.55	65.35–82.37
*sej*	0	0	0.00–0.34
*hla*	90	81.82	73.33–88.53
*hlb*	17	15.45	9.27–23.59
*hld*	98	89.09	81.72–94.23
*hlgAC*	96	87.27	79.57–92.86

**Table 4:** Prevalence and 95% confidence interval (95% C.I.) of virulence gene profiles in 110 strains of *S. aureus* isolated from sheep with mastitis.

Profiles	Genes												Number	Prevalence	95% C.I.
	*sea*	*sec*	*sed*	*seg*	*seh*	*sei*	*sel*	*sej*	*hla*	*hlb*	*hld*	*hlg AC*		%	%
SA1	-	-	-	-	-	-	-	-	-	-	+	+	2	1.82	0.22–6.41
SA2	-	-	-	-	-	-	-	-	-	+	+	+	1	0.91	0.00–4.96
SA3	-	-	-	-	-	-	-	-	+	-	+	-	1	0.91	0.00–4.96
SA4	-	-	-	-	-	-	-	-	+	-	+	+	19	17.27	10.21–24.34
SA5	-	-	-	-	-	-	-	-	+	+	-	+	1	0.91	0.00–4.96
SA6	-	-	-	-	-	-	-	-	+	+	+	+	2	1.82	0.22–5.14
SA7	-	+	-	-	-	-	+	-	-	-	-	-	1	0.91	0.00–4.96
SA8	-	+	-	-	-	-	+	-	-	-	+	-	2	1.82	0.22–6.41
SA9	-	+	-	-	-	-	+	-	-	+	-	-	1	0.91	0.00–4.96
SA10	-	+	-	-	-	-	+	-	-	-	-	+	4	3.64	1.00–9.05
SA11	-	+	-	-	-	-	+	-	-	-	+	+	6	5.45	2.03–11.49
SA12	-	+	-	-	-	-	+	-	-	+	+	+	2	1.82	0.22–6.41
SA13	-	+	-	-	-	-	+	-	+	-	+	-	6	5.45	2.03–11.49
SA14	-	+	-	-	-	-	+	-	+	+	+	-	3	2.73	0.57–7.76
SA15	-	+	-	-	-	-	+	-	+	-	-	+	3	2.73	0.57–7.76
SA16	-	+	-	-	-	-	+	-	+	-	+	+	48	43.64	3420–5342
SA17	-	+	-	-	-	-	+	-	+	+	-	+	1	0.91	0.00–4.96
SA18	-	+	-	-	-	-	+	-	+	+	+	+	5	4.55	1.49–10.29
SA19	-	-	-	-	+	-	-	-	-	-	-	+	1	0.91	0.00–4.96
SA20	-	-	-	+	-	+	-	-	+	+	+	+	1	0.91	0.00–4.96
SA ATCC21	-	-	+	+	-	+	-	+	+	+	+	+			
SA ATCC22	+	+	-	+	-	+	+	-	+	-	+	+			
SA ATCC23	-	+	-	+	+	+	+	-	+	-	+	+			

**Table 5:** Prevalence and 95% confidence interval (95% C.I.) of enterotoxin gene profiles in 110 strains of *S. aureus* isolated from sheep with mastitis.

Profiles	Genes								Number	Prevalence	95% C.I.
	*sea*	*sec*	*sed*	*seg*	*seh*	*sei*	*sel*	*sej*		%	%
SAET1	-	-	-	-	-	-	-	-	26	23.6	16.06–32.68
SAET2	-	+	-	-	-	-	-	+	82	74.6	65.35–82.37
SAET3	-	-	-	-	+	-	-	-	1	0.91	0.00–4.96
SAET4	-	-	-	+	-	+	-	-	1	0.91	0.00–4.96
SA ATCC21	-	-	+	+	-	+	+	-			
SA ATCC22	+	+	-	+	-	+	-	+			
SA ATCC23	-	+	-	+	+	+	-	+			

**Table 6:** Prevalence and 95% confidence interval (95% C.I.) of hemolysin gene profiles in 110 strains of *S. aureus* isolated from sheep with mastitis.

Profiles	Genes				Number	Prevalence	95% C.I.
	*hla*	*hlb*	*hld*	*hlgAC*		%	%
SAEM1	-	-	-	-	1	0.91	0.00–4.96
SAEM2	-	-	+	-	2	1.82	0.22–6.41
SAEM3	-	+	-	-	1	0.91	0.00–4.96
SAEM4	-	-	-	+	5	4.55	1.49–10.29
SAEM5	-	-	+	+	8	7.27	3.19–13.83
SAEM6	-	+	+	+	3	2.73	0.57–7.76
SAEM7	+	-	+	-	7	6.36	2.60–12.67
SAEM8	+	+	+	-	3	2.73	0.57–7.76
SAEM9	+	-	-	+	3	2.73	0.57–7.76
SAEM10	+	-	+	+	67	60.91	51.14–70.07
SAEM11	+	+	-	+	2	1.82	0.22–6.41
SAEM12	+	+	+	+	8	7.27	3.19–13.83
SA ATCC21	+	+	+	+			
SA ATCC22	+	-	+	+			
SA ATCC23	+	-	+	+			

**Table 7:** Prevalence and 95% confidence interval (95% C.I.) of gene profiles and hemolysin profiles of *S. aureus* isolated from milk samples with subclinical mastitis in the same farm.

Gene Profile	Hemolysin Profile	Number	Prevalence	95% C.I.
SA7	SAEM1	1	7.14	0.18–33.87
SA9	SAEM3	1	7.14	0.18–33.87
SA10	SAEM4	3	21.43	4.66–50.80
SA11	SAEM5	2	14.29	1.78–42.81
SA16	SAEM10	6	42.86	17.66–71.14
SA17	SAEM11	1	7.14	0.18–33.87

## Data Availability

Data are contained within the article and Supplementary Materials.

## Supplementary Materials

The following supporting information can be downloaded at https://www.mdpi.com/article/10.3390/ani14223172/s1, Table S1. *Staphylococcus aures* stains isolated from sheep mastitis milk used in the manuscript: coordinates of the place, date of isolation, and the virulence genes’ presence (present: +, assent: −).
