# Supplementary material for: An Investigation of Virulence Genes of Staphylococcus aureus in Autologous Vaccines Against Sheep Mastitis
Source: Animals (Basel). 2024 Nov 6;14(22):3172. doi: 10.3390/ani14223172 (PMC11591023; doi:10.3390/ani14223172)
Supplement: Supplementary file 1 [file animals-14-03172-s001.zip › animals-3262300-supplementary.pdf]

Table S1: *Staphylococcus aures* stains isolated from sheep mastitis milk used in the manuscript: coordinates of the place, date of isolation and the virulence genes' presence (present: +, assent: -)

Progressive number	Coordinates		Date of sampling	Gene presence											
	Latitude	Longitude		*sea*	*sec*	*sed*	*seg*	*seh*	*sei*	*sel*	*sej*	*hla*	*hlb*	*hld*	*hlgAC*
1	43,151281	11,445892	14/03/2019	-	+	-	-	-	-	+	-	+	-	+	+
2	43,118332	11,554742	18/01/2019	-	+	-	-	-	-	+	-	+	+	+	+
3	43,268580	11,040711	25/02/2019	-	+	-	-	-	-	+	-	+	-	+	+
4	42,828450	11,188080	01/06/2019	-	-	-	-	-	-	-	-	+	+	-	+
5	42,953329	11,183539	11/11/2019	-	+	-	-	-	-	+	-	-	-	+	-
6	42,811920	11,217270	01/12/2019	-	+	-	-	-	-	+	-	-	-	-	+
7	42,550833	11,204722	14/12/2019	-	+	-	-	-	-	+	-	+	-	+	+
8	42,581944	11,204167	03/07/2019	-	+	-	-	-	-	+	-	+	-	+	+
9	42,946161	11,321724	29/01/2019	-	+	-	-	-	-	+	-	-	-	+	+
10	42,847037	11,464886	05/02/2019	-	+	-	-	-	-	+	-	+	-	+	+
11	42,480000	11,468056	06/02/2019	-	-	-	-	-	-	-	-	+	-	+	+
12	42,766430	11,366020	13/02/2019	-	+	-	-	-	-	+	-	+	+	+	-
13	42,814117	11,407574	23/04/2019	-	+	-	-	-	-	+	-	+	-	+	+
14	43,296775	11,497958	18/10/2019	-	+	-	-	-	-	+	-	+	-	-	+
15	43,209723	11,652389	10/12/2019	-	-	-	-	-	-	-	-	+	-	+	+
16	43,383749	11,065314	07/01/2019	-	+	-	-	-	-	+	-	+	-	+	+
17	42,884700	11,796914	31/01/2019	-	+	-	-	-	-	+	-	+	-	+	+
18	43,075884	11,849067	06/02/2019	-	+	-	-	-	-	+	-	+	-	+	+
19	43,282899	11,514039	07/02/2019	-	-	-	-	-	-	-	-	+	+	+	+
20	43,199567	11,386346	26/03/2019	-	+	-	-	-	-	+	-	+	-	+	+
21	42,600110	11,406770	08/04/2019	-	+	-	-	-	-	+	-	+	-	+	+
22	43,327834	11,009016	29/04/2019	-	-	-	+	-	+	-	-	+	+	+	+
23	43,255559	11,437327	08/05/2019	-	-	-	-	-	-	-	-	+	-	+	+
24	43,127107	11,411873	17/10/2019	-	+	-	-	-	-	+	-	+	-	+	+
25	43,267142	11,442619	06/11/2019	-	-	-	-	-	-	-	-	+	-	+	+
26	42,594010	11,224160	29/11/2019	-	-	-	-	-	-	-	-	+	-	+	+
27	42,594010	11,224160	05/12/2019	-	-	-	-	-	-	-	-	+	-	+	+
28	42,848001	11,406442	06/12/2019	-	+	-	-	-	-	+	-	+	+	+	+
29	43,279463	11,533443	27/01/2020	-	-	-	-	-	-	-	-	-	+	+	+
30	43,328567	11,018716	07/02/2020	-	-	-	-	-	-	-	-	+	-	+	+
31	42,733333	11,238611	11/02/2020	-	-	-	-	-	-	-	-	+	-	+	+
32	43,143090	11,776926	17/02/2020	-	-	-	-	-	-	-	-	+	-	+	+
33	43,209723	11,652389	26/02/2020	-	-	-	-	-	-	-	-	+	-	+	+
34	42,583611	11,352778	30/03/2020	-	+	-	-	-	-	+	-	+	-	+	+
35	42,544520	11,191360	12/05/2020	-	+	-	-	-	-	+	-	-	+	+	+
36	42,930540	11,353970	13/05/2020	-	+	-	-	-	-	+	-	+	-	+	+
37	42,703000	11,394100	22/05/2020	-	+	-	-	-	-	+	-	+	-	+	+
38	42,930540	11,353970	03/06/2020	-	+	-	-	-	-	+	-	+	-	+	+
39	42,673460	11,402730	08/07/2020	-	-	-	-	-	-	-	-	+	-	+	+
40	43,268277	11,516468	25/08/2020	-	+	-	-	-	-	+	-	+	+	+	+
41	42,588929	11,517380	25/08/2020	-	+	-	-	-	-	+	-	+	+	+	-

42	42,428860	11,474190	21/10/2020	-	-	-	-	-	-	-	-	+	+	+	+
43	42,767130	11,263100	17/11/2020	-	+	-	-	-	-	+	-	+	-	+	+
44	43,088929	11,822977	18/12/2020	-	-	-	-	-	-	-	-	+	-	+	-
45	43,146600	10,811150	18/12/2020	-	-	-	-	-	-	-	-	+	-	+	+
46	42,470917	11,612477	18/01/2021	-	+	-	-	-	-	+	-	+	-	+	+
47	42,663110	11,150830	27/01/2021	-	+	-	-	-	-	+	-	+	-	+	+
48	43,229273	11,734386	28/01/2021	-	+	-	-	-	-	+	-	+	-	+	+
49	43,174864	11,130379	05/02/2021	-	+	-	-	-	-	+	-	+	+	+	+
50	43,134588	11,123607	08/03/2021	-	+	-	-	-	-	+	-	-	-	+	+
51	42,779170	11,364380	17/03/2021	-	+	-	-	-	-	+	-	+	-	+	+
52	42,538870	11,435460	23/03/2021	-	+	-	-	-	-	+	-	+	-	+	+
53	42,684620	11,657080	26/03/2021	-	-	-	-	-	-	-	-	+	-	+	+
54	42,779170	11,364380	08/04/2021	-	+	-	-	-	-	+	-	+	-	+	-
55	42,845350	11,233220	27/04/2021	-	+	-	-	-	-	+	-	+	-	+	-
56	42,808966	11,456845	06/07/2021	-	+	-	-	-	-	+	-	+	-	+	+
57	42,703000	11,394100	07/09/2021	-	+	-	-	-	-	+	-	+	-	+	+
58	42,560120	11,442160	21/09/2021	-	+	-	-	-	-	+	-	+	-	+	+
59	42,767130	11,263100	26/10/2021	-	+	-	-	-	-	+	-	+	-	+	+
60	43,135833	11,780987	27/10/2021	-	-	-	-	-	-	-	-	-	-	+	+
61	42,571370	11,556820	03/11/2021	-	+	-	-	-	-	+	-	+	-	+	+
62	42,862766	11,749387	16/11/2021	-	-	-	-	-	-	-	-	+	-	+	+
63	42,538870	11,435460	17/11/2021	-	+	-	-	-	-	+	-	+	-	+	+
64	42,759910	11,170430	25/11/2021	-	-	-	-	-	-	-	-	+	-	+	+
65	42,656690	11,179630	02/12/2021	-	+	-	-	-	-	+	-	+	-	+	-
66	42,608889	11,412778	29/12/2021	-	-	-	-	-	-	-	-	+	-	+	+
67	43,163808	11,626440	18/01/2022	-	+	-	-	-	-	+	-	+	-	+	+
68	43,397280	11,295933	31/01/2022	-	+	-	-	-	-	+	-	+	-	+	+
69	43,082288	11,714257	08/03/2022	-	+	-	-	-	-	+	-	+	-	-	+
70	42,894670	11,050420	16/03/2022	-	+	-	-	-	-	+	-	+	-	+	+
71	43,373696	11,264964	21/03/2022	-	+	-	-	-	-	+	-	+	-	+	+
72	42,878404	11,755089	30/03/2022	-	-	-	-	-	-	-	-	+	-	+	+
73	42,811350	11,303060	27/04/2022	-	+	-	-	-	-	+	-	+	-	+	+
74	42,602140	11,460810	27/05/2022	-	+	-	-	-	-	+	-	+	-	+	+
75	43,258361	11,596306	30/07/2022	-	+	-	-	-	-	+	-	-	-	+	+
76	42,558050	11,435690	17/10/2022	-	+	-	-	-	-	+	-	-	+	+	+
77	42,583611	11,352778	26/10/2022	-	+	-	-	-	-	+	-	+	-	+	+
78	42,755340	11,338856	10/11/2022	-	-	-	-	-	-	-	-	+	-	+	+
79	42,902090	11,045090	23/11/2022	-	+	-	-	-	-	+	-	+	-	+	+
80	43,074423	10,734209	29/12/2022	-	+	-	-	-	-	+	-	+	-	+	+
81	42,643410	11,274410	30/12/2022	-	+	-	-	-	-	+	-	+	-	+	+
82	42,515000	11,445833	04/01/2023	-	+	-	-	-	-	+	-	+	-	+	-
83	43,129431	11,789510	10/01/2023	-	+	-	-	-	-	+	-	+	-	-	+
84	42,942273	11,113637	11/01/2023	-	-	-	-	-	-	-	-	+	-	+	+
85	42,703060	11,640000	30/01/2023	-	+	-	-	-	-	+	-	+	-	+	+
86	43,014970	11,743140	16/02/2023	-	-	-	-	+	-	-	-	-	-	-	+

87	43,246810	11,035815	07/03/2023	-	+	-	-	-	-	+	-	-	-	+	-
88	42,791250	11,314880	17/03/2023	-	+	-	-	-	-	+	-	+	-	+	+
89	42,684722	11,768889	20/03/2023	-	+	-	-	-	-	+	-	+	+	+	-
90	43,304264	11,461131	21/03/2023	-	-	-	-	-	-	-	-	-	-	+	+
91	42,583260	11,200840	11/04/2023	-	+	-	-	-	-	+	-	+	+	+	+
92	43,094651	11,754868	27/06/2023	-	+	-	-	-	-	+	-	+	-	+	+
93	42,571370	11,556820	04/11/2020	-	+	-	-	-	-	+	-	+	-	+	-
94	42,571370	11,556820	04/11/2020	-	+	-	-	-	-	+	-	+	-	+	-
95	42,571370	11,556820	16/11/2020	-	+	-	-	-	-	+	-	+	-	+	+
96	42,639650	11,385530	25/02/2021	-	+	-	-	-	-	+	-	-	-	+	+
97	42,538870	11,435460	17/11/2021	-	+	-	-	-	-	+	-	+	-	+	+
98	42,538870	11,435460	17/11/2021	-	+	-	-	-	-	+	-	-	-	+	+
99	42,538870	11,435460	17/11/2021	-	+	-	-	-	-	+	-	+	-	+	+
100	42,538870	11,435460	17/11/2021	-	+	-	-	-	-	+	-	+	-	+	+
101	42,538870	11,435460	17/11/2021	-	+	-	-	-	-	+	-	+	-	+	+
102	42,538870	11,435460	17/11/2021	-	+	-	-	-	-	+	-	-	-	-	+
103	42,538870	11,435460	17/11/2021	-	+	-	-	-	-	+	-	-	-	-	+
104	42,538870	11,435460	17/11/2021	-	+	-	-	-	-	+	-	-	+	-	-
105	42,538870	11,435460	17/11/2021	-	+	-	-	-	-	+	-	+	+	-	+
106	42,538870	11,435460	17/11/2021	-	+	-	-	-	-	+	-	-	-	-	-
107	42,538870	11,435460	17/11/2021	-	+	-	-	-	-	+	-	-	-	-	+
108	42,538870	11,435460	17/11/2021	-	+	-	-	-	-	+	-	+	-	+	+
109	42,538870	11,435460	17/11/2021	-	+	-	-	-	-	+	-	-	-	+	+
110	42,538870	11,435460	17/11/2021	-	+	-	-	-	-	+	-	+	-	+	+
